# Effect of lung ultrasound-guided fluid deresuscitation on duration of ventilation in intensive care unit patients (CONFIDENCE): protocol for a multicentre randomised controlled trial

**DOI:** 10.1186/s13063-023-07171-w

**Published:** 2023-03-24

**Authors:** Siebe G. Blok, Amne Mousa, Michelle G. Brouwer, Harm-Jan de Grooth, Ary Serpa Neto, Michiel J. Blans, Sylvia den Boer, Tom Dormans, Henrik Endeman, Timo Roeleveld, Harm Scholten, Eline R. van Slobbe-Bijlsma, Erik Scholten, Hugo Touw, Fleur Stefanie L. I. M. van der Ven, Evert-Jan Wils, David J. van Westerloo, Leo M. A. Heunks, Marcus J. Schultz, Frederique Paulus, Pieter R. Tuinman

**Affiliations:** 1 Department of Intensive Care, Amsterdam UMC, University of Amsterdam, Meibergdreef 9, Amsterdam, Netherlands; 2 Amsterdam Leiden Intensive care Focused Echography (ALIFE, www.alifeofpocus.com ), Amsterdam, The Netherlands; 3 Amsterdam Leiden Intensive care Focused Echography (ALIFE, www.alifeofpocus.com ), Leiden, The Netherlands; 4 Department of Intensive Care, Amsterdam Cardiovascular Sciences, Amsterdam UMC, Vrije Universiteit Amsterdam, De Boelelaan, 1117 Amsterdam, Netherlands; 5 Department of Critical Care Medicine, Hospital Israelita Albert Einstein, Sao Paulo, Brazil; 6 Australian and New Zealand Intensive Care Research Centre (ANZIC-RC), School of Public Health and Preventive Medicine, Monash University, Melbourne, Australia; 7 Department of Critical Care, Melbourne Medical School, University of Melbourne, Austin Hospital, Melbourne, Australia; 8 Department of Intensive Care, Austin Hospital, Melbourne, Australia; 9 Department of Intensive Care, Rijnstate Hospital, Arnhem, Netherlands; 10 Department of Intensive Care, Spaarne Gasthuis, Haarlem, Hoofddorp, Netherlands; 11 Department of Intensive Care, Zuyderland Medical Centre, Heerlen, Netherlands; 12 Department of Intensive Care, Zuyderland Medical Centre, Sittard-Geleen, Netherlands; 13 Department of Intensive Care, Erasmus MC, Rotterdam, Netherlands; 14 Department of Intensive Care, Amstelland Hospital, Amstelveen, Netherlands; 15 Department of Intensive Care, Catharina Hospital, Eindhoven, Netherlands; 16 Department of Intensive Care, TerGooi MC, Hilversum, Netherlands; 17 Department of Intensive Care, St. Antonius Hospital, Nieuwegein, Utrecht, Netherlands; 18 Department of Intensive Care, Radboud University Medical Centre, Nijmegen, Netherlands; 19 Department of Intensive Care, Rode Kruis Hospital, Beverwijk, Netherlands; 20 Department of Intensive Care, Franciscus Gasthuis & Vlietland, Rotterdam, Netherlands; 21 Department of Intensive Care, Leiden University Medical Centre, Leiden, Netherlands; 22 Center of Expertise Urban Vitality, Faculty of Health, Amsterdam University of Applied Sciences, Amsterdam, The Netherlands

**Keywords:** Pulmonary oedema, Deresuscitation, Invasive ventilation, Critical care, Lung ultrasound, Randomised controlled trial

## Abstract

**Background:**

Fluid therapy is a common intervention in critically ill patients. It is increasingly recognised that deresuscitation is an essential part of fluid therapy and delayed deresuscitation is associated with longer invasive ventilation and length of intensive care unit (ICU) stay. However, optimal timing and rate of deresuscitation remain unclear. Lung ultrasound (LUS) may be used to identify fluid overload. We hypothesise that daily LUS-guided deresuscitation is superior to deresuscitation without LUS in critically ill patients expected to undergo invasive ventilation for more than 24 h in terms of ventilator free-days and being alive at day 28.

**Methods:**

The “effect of lung ultrasound-guided fluid deresuscitation on duration of ventilation in intensive care unit patients” (CONFIDENCE) is a national, multicentre, open-label, randomised controlled trial (RCT) in adult critically ill patients that are expected to be invasively ventilated for at least 24 h. Patients with conditions that preclude a negative fluid balance or LUS examination are excluded. CONFIDENCE will operate in 10 ICUs in the Netherlands and enrol 1000 patients. After hemodynamic stabilisation, patients assigned to the intervention will receive daily LUS with fluid balance recommendations. Subjects in the control arm are deresuscitated at the physician’s discretion without the use of LUS. The primary endpoint is the number of ventilator-free days and being alive at day 28. Secondary endpoints include the duration of invasive ventilation; 28-day mortality; 90-day mortality; ICU, in hospital and total length of stay; cumulative fluid balance on days 1–7 after randomisation and on days 1–7 after start of LUS examination; mean serum lactate on days 1–7; the incidence of reintubations, chest drain placement, atrial fibrillation, kidney injury (KDIGO stadium ≥ 2) and hypernatremia; the use of invasive hemodynamic monitoring, and chest-X-ray; and quality of life at day 28.

**Discussion:**

The CONFIDENCE trial is the first RCT comparing the effect of LUS-guided deresuscitation to routine care in invasively ventilated ICU patients. If proven effective, LUS-guided deresuscitation could improve outcomes in some of the most vulnerable and resource-intensive patients in a manner that is non-invasive, easy to perform, and well-implementable.

**Trial registration:**

ClinicalTrials.gov NCT05188092. Registered since January 12, 2022

**Supplementary Information:**

The online version contains supplementary material available at 10.1186/s13063-023-07171-w.

## Background

Fluid resuscitation is a ubiquitous intervention in critically ill patients. Current international guidelines recommend aggressive fluid resuscitation in hemodynamically unstable critically ill patients to restore intravascular volume and maintain organ perfusion [1, 2]. However, even in healthy, hypovolemic subjects, only 40% of a fluid bolus remains in the vasculature after 15 min [3]. This fluid extravasation is exacerbated in critical illness, often leading to tissue oedema [4].

Pulmonary oedema is a strong and independent predictor of death [4–8]. Furthermore, delayed fluid deresuscitation is independently associated with a longer need for invasive ventilation and consequently increased length of stay in the intensive care unit (ICU) [9, 10]. The association between a positive cumulative fluid balance (CFB), pulmonary oedema, and poor outcomes is well recognised [4–10], whereas evidence of beneficial outcomes when employing more aggressive deresuscitation strategies is growing [1, 11, 12]. No guidelines on deresuscitation are available, and clinical practice is highly variable [13]. The presence of pulmonary oedema is a sign that the limit of fluid tolerance has been reached or exceeded. Therefore, if pulmonary oedema is present in the context of hemodynamic stability or improvement, active fluid removal (deresuscitation) initiated as soon as possible may lead to faster pulmonary recovery and better outcomes.

However, recognising pulmonary oedema can be challenging. The use of pulmonary artery catheters or pulse contour cardiac output measurement techniques can provide guidance, but these techniques are invasive, complex, and expensive [14]. Chest radiography is inexpensive and easily performed but has insufficient accuracy to detect pulmonary oedema in critically ill patients and exposes the patient to radiation [15].

**Fig. 1:**
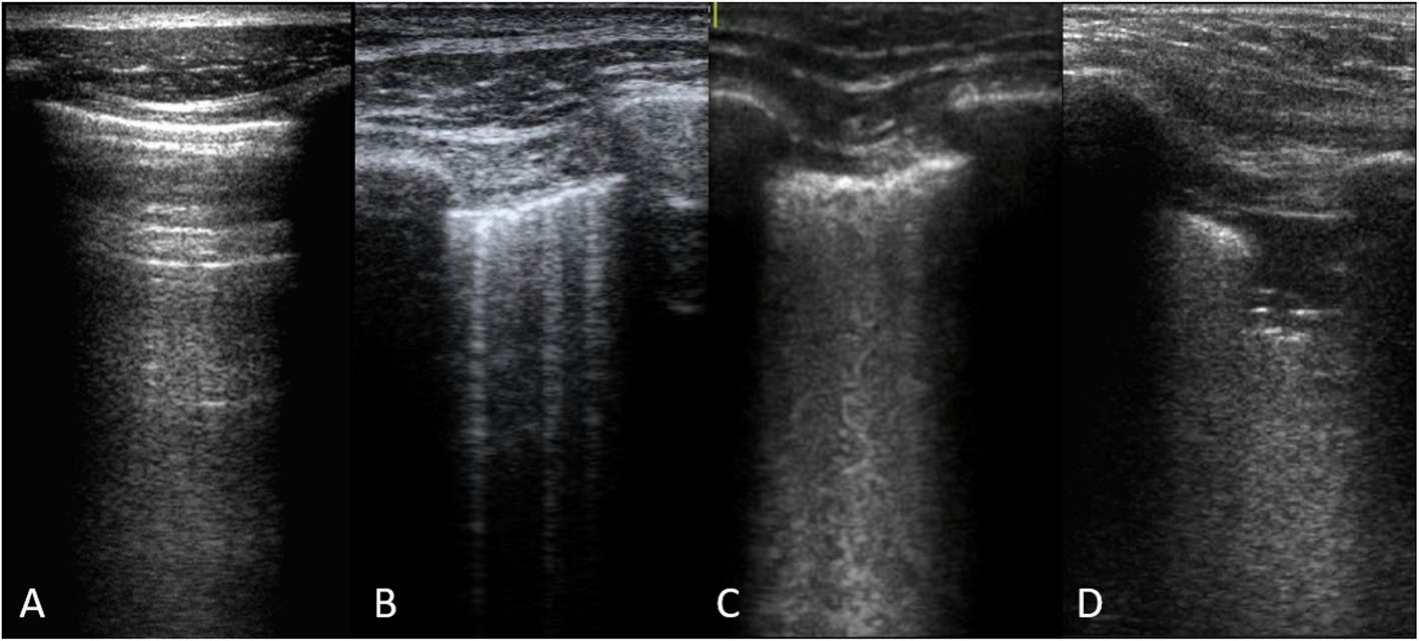
LUS images with increasing loss of aeration. **A** Normally aerated lung, referred to as an A-profile. **B** Moderate loss of aeration leading to a B1-profile with 3 discrete B-lines. **C** Severe loss of aeration leading to a B2-profile with confluent B-lines. **D** Complete loss of aeration leading to consolidation

In the last decade, lung ultrasound (LUS) is increasingly used for the clinical assessment of critically ill patients [15]. LUS is a simple, safe, and non-invasive bedside imaging tool that has a high diagnostic accuracy for pulmonary fluid overload [16, 17]. LUS findings consist of both artefacts (A- and B-lines) and real images (consolidations and pleural effusions) (Fig. 1). While the presence of so-called ‘A–lines’ suggests normal lung tissue, the presence of three or more so-called ‘B–lines’ indicates abnormal lung tissue, most frequently because of pulmonary oedema. LUS of the anterior, lateral, and posterior chest regions correlates well with extravascular lung water [16, 18]. Furthermore, even small amounts of pleural effusion are readily detected by LUS. Consequently, LUS could potentially guide decisions regarding deresuscitation, as the presence of significant extravascular lung water or (new) pleural effusion should trigger deresuscitation in hemodynamically stable patients.

Randomised controlled trials (RCT) consistently show that guiding fluid management with LUS is associated with a lower CFB [19]. In addition, LUS frequently changes patient treatment in the emergency department, ICU and medical ward [20]. However, previous research was limited by a lack of power and did not focus on clinical endpoints. Thus, while earlier findings suggest that the use of LUS to guide deresuscitation might improve patient outcomes, this has not been fully elucidated. We hypothesise that in critically ill patients expected to undergo invasive ventilation for more than 24 h, daily LUS-guided deresuscitation is superior to deresuscitation without LUS in terms of ventilator-free days and being alive at day 28.

## Methods

### Objectives and design

The primary aim of this RCT is to evaluate if LUS-guided deresuscitation leads to a shorter period of invasive ventilation and/or a reduction in mortality. We hypothesise that daily LUS leads to timely detection of pulmonary oedema which triggers removal of excess fluid, leading to a more appropriate fluid status which reduces duration of invasive ventilation and might also reduce mortality. In addition, we hypothesise that LUS-guided deresuscitation does not lead to an increased incidence of acute kidney injury or increased ICU length of stay. This study is an investigator-initiated, national, multicentre, parallel-group, open-label RCT in critically ill and invasively ventilated adult patients admitted to the ICUs of participating hospitals. Patients will be randomly assigned in a 1:1 ratio to either LUS-guided fluid deresuscitation or usual care. The study will enrol 1000 patients in total and will be executed in ten ICUs in academic and community hospitals in The Netherlands. A list of currently participating centres is available at https://www.clinicaltrials.gov (NCT05188092).

### Study population

Patients admitted to one of the participating ICUs and expected to be under invasive ventilation for longer than 24 h after randomisation will be screened for eligibility and randomised during the first 24 h of invasive ventilation. Patients aged < 18 years at randomisation are not eligible. Patients with conditions in which a negative CFB is discouraged, such as subarachnoid bleeding and severe rhabdomyolysis; patients in which LUS cannot be performed or correctly interpreted, for example, due to morbid obesity, chest-wall abnormalities or pre-existing interstitial lung disease; severe burns; pregnancy; participation in other interventional trials with similar endpoints; the use of long term home mechanical ventilation; extracorporeal membrane oxygenation; previous participation in this RCT; and neurological conditions that could prolong the duration of invasive ventilation, such as Guillain-Barré syndrome, high spinal cord lesion, amyotrophic lateral sclerosis, multiple sclerosis, and myasthenia gravis are excluded from participation.

### Intervention

Patients in the usual care arm will be deresuscitated at the discretion of the treating physician. Fluid deresuscitation can be guided by several modalities (e.g. physical examination, laboratory tests, chest X-ray and pulse contour cardiac output or pulmonary artery catheter). LUS, however, may not be used to guide fluid deresuscitation in this group.

In the LUS-guided fluid deresuscitation arm, LUS examinations are performed at least once a day when the patient is hemodynamically stable. Hemodynamic stability is defined as mean arterial pressure (MAP) ≥ 65 mmHg (with vasopressor dose clearly decreasing and norepinephrine ≤ 0.2 μg/kg/min), arterial lactate level < 2.5 mmol/L (or < 4 mmol/L and decreased with > 25% in last hours), and no clear signs of hypoperfusion such as mottled skin and capillary refill time > 3 s and/or new oliguria (urine output < 0.3–0.5 ml/kg/h for the previous 6 h). If some of these clinical signs are not related to hypoperfusion, these signs can be disregarded at the discretion of the treating physician. Target MAP can be adjusted in conditions in which 65 mmHg is not sufficient (i.e. history of chronic hypertension). In these cases, the target MAP is at the discretion of the treating physician. 12-region LUS is performed by a trained healthcare provider, and each region is scanned for the presence of B-lines, consolidations, and pleural effusions and scored using the lung ultrasound score (Supplement I) [21]. LUS examinations will be performed daily until discharge from the ICU or until day 28, whichever comes first. Any healthcare provider that is trained in LUS is eligible to perform the LUS. We do not require formal certification, since formal certification is not considered a necessity in regular clinical practice. In centres that are not already using LUS in daily practice, physicians are trained in the use of LUS before the start of the study. These physicians will be tested on LUS interpretation skills before and after training.

**Fig. 2:**
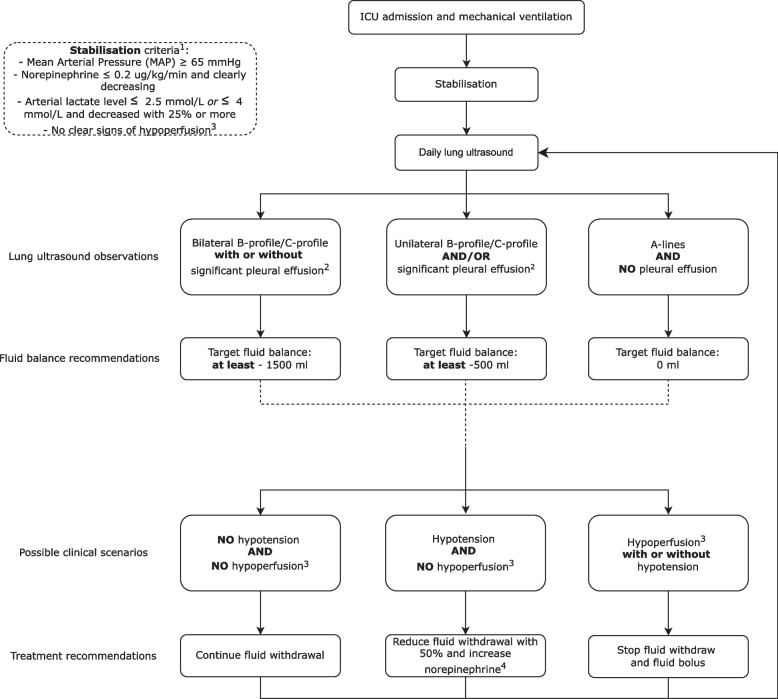
Flowchart of the investigational treatment. LUS is performed daily after stabilisation and a recommendation towards fluid balance for the next 24 h is given based on LUS findings. Insensible loss is not taken into account for the fluid balance. ^1^: if some of these clinical signs are not related to hypoperfusion, these signs can be disregarded at the discretion of the treating physician. ^2^: significant pleural effusion: > 1 cm lateral fields (R3, R4, L3, L4), > 2 cm posterior fields (R5, R6, L5, L6). ^3^: hypoperfusion is defined by new development of mottled skin, capillary refill time > 3 s, or new oliguria (urine output < 0.3–0.5 ml/kg/h for the previous 6 h). ^4^: until norepinephrine > 0.2 μg/kg/min. Fluid boluses can be given if hypotension persists

The results of each LUS examination are categorised into one of three summary findings, each with a distinct treatment recommendation (Fig. 2):

1. LUS suggests substantial pulmonary oedema. This is defined as the presence of a bilateral B-profile (≥ 3 B-lines) or C-profile in anterior or lateral regions (Supplement I). We recommend the clinician to target a negative fluid balance of at least − 1500 ml in the next 24 h.
2. LUS suggests some pulmonary oedema and/or significant pleural effusion. This is defined as the presence of a unilateral B-profile (≥3 B-lines) or C-profile in the anterior or lateral regions, or pleural effusion > 1 cm in lateral regions, or > 2 cm in posterior regions. We recommend the clinician to target a negative fluid balance of at least − 500 ml in the next 24 h.
3. LUS suggests no pulmonary oedema and no pleural effusion. The absence of pulmonary oedema is defined as the absence of a B-profile (< 3 B-lines) or C-profile in anterior or lateral regions. We recommend the clinician to target a neutral fluid balance in the next 24 h.

In between LUS examinations, additional management recommendations are given for the following two scenarios:

1. In the case of hypotension without clear signs of hypoperfusion, fluid withdrawal should be decreased by 50% and a vasopressor infusion is started or increased. Fluid boluses should only be given when vasopressor dosage becomes too high (norepinephrine > 0.2 μg/kg/min).
2. In the case of hypotension with clear signs of hypoperfusion, a small fluid bolus of 250 ml of fluid can be administered and vasopressor infusion should be increased. Fluid administration can be repeated at the discretion of the attending physician.

After extubation, LUS is continued once a day until day 28 or until discharge from the ICU to monitor the reappearance of pulmonary fluid overload or pleural effusion. The treatment algorithm based on LUS outcomes does not change. Clinicians can disregard the recommended CFB target if there is any concern for subject safety.

### Standard procedures beyond LUS

Drainage of the intra-pleural space using chest drains is permitted in both arms. The placement of chest drains is at the discretion of the attending physician and will be performed in keeping with the local guidelines.

Early tracheostomy has no average advantage over late tracheostomy [22]. Therefore, tracheostomy is only to be performed on strict indication and preferably not earlier than 10 days after intubation.

Sedation follows local guidelines in each participating unit. In general, these guidelines favour the use of analgo–sedation over hypno–sedation, the use of bolus over continuous infusion of sedating agents, and the use of sedation scores.

Selective decontamination of the digestive tract is practised in patients who are expected to need invasive ventilation for more than 48 h and/or expected not to be discharged from the ICU within 72 h.

Thrombosis prophylaxis is indicated for all patients who are not already treated with anticoagulants. Thrombosis prophylaxis will be given according to local guidelines.

Fluid therapy is targeted at adequate organ perfusion. Crystalloid and balanced infusions are preferred over colloid infusions. In the case of atrial fibrillation, the continuation of fluid withdrawal is advised. In the event of mild to moderate hypernatremia (145–155 mmol/L), the continuation of fluid withdrawal is advised and the administration of enteral free water and/or intravenous 5% dextrose should be considered. If severe hypernatremia (> 155 mmol/L) occurs, the discontinuation of fluid withdrawal and the administration of enteral free water and/or intravenous 5% dextrose is advised. Rising serum creatinine levels without signs of hypoperfusion should not lead to discontinuation of fluid withdrawal.

Weaning is standardised in both groups, as weaning directly influences the primary outcome measure. The ventilator can be switched to partially supported ventilation mode at any moment the attending nurse or physician consider the patient’s respiratory drive sufficient to breathe with partially supported ventilation. At least once a day assessment of the ability to breathe without mechanical ventilation is required as soon as (1) FiO_2_ ≤ 0.5 or (2) when the PEEP (≤ 10 cm H_2_O) and FiO_2_ are lower than the day before [23]. A patient is assumed to be ready for extubation when the patient is responsive and cooperative, has an adequate cough reflex, a PaO_2_/FiO_2_ of > 200 mmHg with FiO_2_ < 50% and PEEP ≤ 10 cm H_2_O, a respiratory rate between 8 and 30 per minute with a pressure support level < 10 cm H_2_O, and is without signs of respiratory distress. Furthermore, patients should be hemodynamically stable with a systolic blood pressure between 80 and 160 mmHg and a heart rate between 40 and 130 beats per minute without uncontrolled arrhythmia or the use of high-dose vasopressors (norepinephrine > 0.2 μg/kg/min). Finally, core body temperature must be higher than 36.0° Celsius and lower than 38.5° Celsius. If a patient becomes dyspnoeic or hypoxemic after extubation, both non–invasive ventilation and high-flow nasal cannula are permitted as the first line of therapy.

### Endpoints

The primary endpoint is the number of ventilator-free days and alive at day 28 (VFD-28), defined as the number of days from day 1 to day 28 during which the patient is alive and breathes without assistance of the mechanical ventilator. The period of unassisted breathing should last at least 24 consecutive hours.

Secondary endpoints include the duration of invasive ventilation; 28-day mortality; 90-day mortality (ICU, in hospital and overall); length of stay (ICU and in hospital); CFB on days 1–7 after randomisation and on days 1–7 after start of LUS examination; mean serum lactate on days 1–7; the incidence of reintubations, chest drain placement, atrial fibrillation, kidney injury (KDIGO stadium ≥ 2), and hypernatremia; the use of invasive hemodynamic monitoring, and chest-X-ray; and quality of life at day 28. Detailed definitions of all endpoints are found in Supplement II.

### Participant timeline

**Fig. 3:**
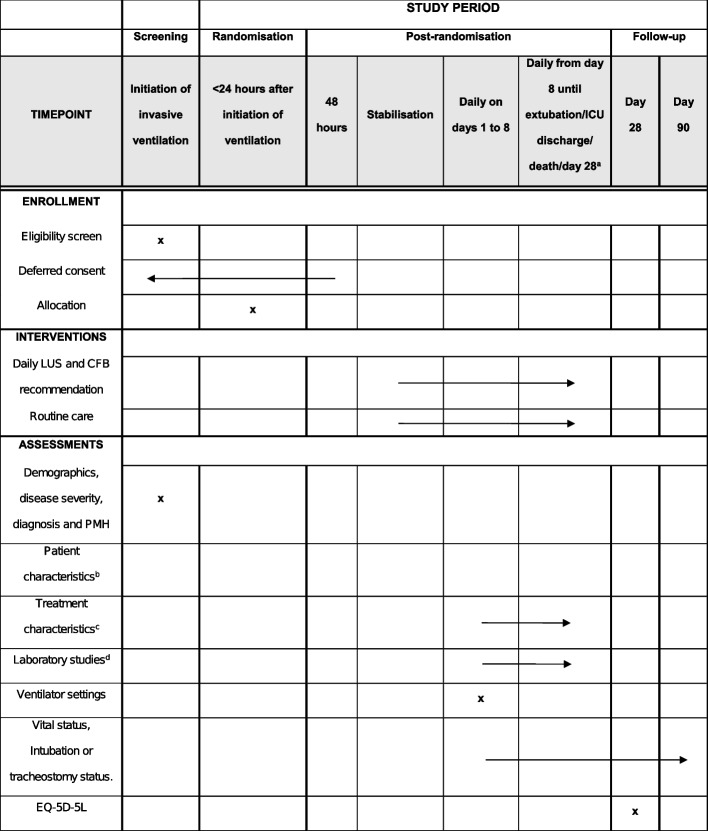
Schedule of enrolment, interventions, and assessments. ^a^: whatever comes first. ^b^: heart rate, mean arterial pressure, Glasgow Coma Scale, new-onset atrial fibrillation. ^c^: CFB, fluid infusion, diuresis, use of vasopressors or inotropes, diuretic use and dosage, blood product transfusions, hemodialysis or hemofiltration. ^d^: arterial blood gas analysis; creatinine, urea and bilirubin plasma levels; thrombocyte count. PMH, past medical history

The participant timeline is shown in Fig. 3.

### Deferred consent

Potential participants in this study will be included using deferred informed consent. As mechanically ventilated patients are generally not able to give informed consent, deferred consent will be requested from the legal representative. Since postponing the start of deresuscitation may influence the trial outcomes, the ethical review board (METC) has judged that proxy consent can be deferred while randomisation and the initiation of therapy take place. Proxy consent can be deferred by no more than 48 hours, by which time all study procedures will be stopped if proxy (or patient) consent has not been obtained. Definite informed consent is asked from the patient, at the moment the patient is awake and able to judge on their situation properly. If a patient declines consent, all collected study data on this patient will be destroyed. In addition, participants are free to leave the study at any time upon request from the legal representative or patient. Participants are asked on the informed consent form if they consent to use the collected data in ancillary studies.

Informed consent will be obtained only by researchers who are GCP certified to perform research under the Dutch Medical Research Involving Human Subjects Act (WMO) or are in the process of obtaining certification, in full compliance with Dutch regulations. Furthermore, those that obtain informed consent will be trained in the study protocol.

### Recruitment

Subjects will be recruited from approximately 10 ICUs in both community and academic hospitals. Approximately 100 participants will be enrolled per centre. The number of inclusions per centre will be recorded and monitored. The amount of subjects per centre is not predefined and, as such, larger volume ICUs can recruit more subjects to compensate for slower recruitment in other centres. If recruitment is not according to schedule, additional ICUs will be approached to join the study thereby expediting participant recruitment.

### Minimisation of bias

Local investigators perform randomisation using the randomisation tool in Castor Electronic Data Capture (EDC). The allocation sequence is generated by Castor EDC, stratified by centre and uses permuted blocks with variable sizes and a maximum of 10. Allocation concealment is ensured by Castor EDC until the participant is randomised. Inherently to the type of intervention, blinding of the caregivers is not possible as the intervention is used for guiding management. Data analysis, however, will be performed blinded for the study intervention. The Standard Protocol Items: Recommendations for Interventional Trials (SPIRIT) 2013 Checklist is supplied as Supplement III.

### Gathering and handling of data

Data is obtained from the electronic patient record and entered directly into the electronic case report form (eCRF) by the investigators. The eCRF was designed by experienced scientific staff specifically for this study and was extensively tested before finalisation. The eCRF has built-in data validation to alert investigators to implausible or incomplete data and prevent the input of impossible data. A list of all data collected is found in Supplement IV. For assessing health-related quality of life 28 days after inclusion, we employ the validated and commonly used EQ-5D-5L questionnaire [24]. This questionnaire is made available in Dutch as well as English. Follow up after 28 and 90 days (with a visiting window of 2 weeks after day 28 and day 90) will be performed via telephone if the subject is discharged from the hospital, or in person if the participant is still admitted.

We expect minimal losses to follow up for the primary endpoint. To further minimise this loss, the following procedures are in place: transferral of study subjects is discouraged at all study sites, unless essential for patient care; contact information for the researchers and an independent expert is supplied on the patient information page that they receive before providing informed consent; legal representatives are encouraged to contact the researchers if they have any questions and researchers are reminded of follow-up by the usage of a subject identification log in which the date of follow-up is noted for every subject.

All patients will be addressed by a random patient identification code, not based on any personal data. The codebook will be stored digitally and on paper. The paper version will be stored behind a lock and the digital form will be encrypted with a double password. All handling of personal data will comply with the Dutch Personal Data Protection Act. The principal investigator, coordinating investigators, healthcare inspectorate, monitors, and auditors will have access to the data and documents. After database close-out, the codebook will be destroyed. All other data will be stored for the length of the study and 15 years afterwards, for further publication.

Trial results will be published in key journals in the field of critical care and presented at international conferences. The publication will adhere to the principles as described by the Central Committee on Research Involving Human Subjects [25]. Those that contributed significantly to trial design and conduct will be authors on the final paper.

### Sample size calculation

The sample size was computed using R statistics version 3.0.2 with the gsDesign package and was based on the assumption that LUS-guided deresuscitation is associated with at least 2 day increase of VFD-28. We consider a 2 day increase of VFD-28 to be clinically relevant and that this would justify the effort of performing LUS examination. Assuming a mean (± SD) number of VFD-28 of 13 ± 11 days [26, 27] we estimate that a sample of 1000 patients (500 per group) is needed to have 80% power, at a two-tailed significance level of 0.05, to detect a mean between-group difference of 2 VFD-28, and allow an anticipated dropout rate of 5%.

### Statistical analysis

A detailed statistical analysis plan will be finalised and made available before the closure of the database. The primary statistical analysis will be based on the intention-to-treat principle. Subjects are analysed according to their assigned treatment arms, except for those who are withdrawn due to lack of deferred informed consent. The goal of the primary analysis is to quantify the effect of LUS-guided deresuscitation strategy vs. routine care on the number of VFD-28. Continuous normally distributed variables will be expressed by their mean and standard deviation or when not normally distributed as medians and their interquartile ranges. Categorical variables will be expressed as n (%). Multiple imputation may be used to handle missing data.

Secondary analyses include analysis of patients who fulfilled the Berlin criteria of ARDS versus patients who did not and patients diagnosed with sepsis versus patients not diagnosed with sepsis. In addition to the intention-to-treat, we will also perform a per-protocol analysis. This analysis will only consider subjects that were treated in accordance with their randomisation arm. No interim analyses towards the primary endpoint will be done.

### Trial organisation

The coordinating centre and sponsor is the Amsterdam UMC. The Amsterdam UMC is responsible for funding, the study design and management and analysis, and the interpretation and publishing of the data. The steering committee consists of four principal investigators, two coordinating investigators, two statisticians, and the local investigators at participating study sites.

The data safety monitoring board (DSMB) consists of three renowned independent intensivists with expertise in clinical trials in the ICU and one independent statistician. The DSMB will meet every 6 months. The responsibilities of the DSMB include monitoring the safety endpoints and monitoring protocol compliance of both treatment strategies by examining the use of LUS. Furthermore, the DSMB will review the overall status of the study: number of patients enrolled overall and, in each centre, adherence to the protocol overall and stratified by centre. Events that can be considered serious adverse events in this critically ill population are death and renal replacement therapy. Blinded tables showing the incidence of these serious adverse events are sent to the METC and DSMB periodically. If any unexpected (serious) adverse events occur that might be related to the study intervention, these will also be reported to the METC and DSMB. The DSMB can advise the sponsor to halt the trial.

An independent monitor provided by the clinical monitoring centre of the Amsterdam UMC will oversee the study, following the approved monitoring plan. This monitor will perform on-site as well as remote surveillance for all involved sites. Monitoring responsibilities include verifying inclusion rate, proper documentation and execution of informed consent, and proper use of inclusion and exclusion criteria. The independent monitor also reviews integrity and completeness of outcome data in the eCRF.

Any modification to the protocol or supporting materials must be communicated to the METC. Any modification that is likely to significantly affect the safety or physical or mental integrity of participants, the scientific value of the study, the conduct of the trial, or the quality or safety of any intervention used in the trial will have to be evaluated by the METC before being implemented. Protocol amendments that directly affect patient participation will be noted on the informed consent form and subjects or legal representatives will be asked to re-consent, except for subjects that had already completed follow-up.

## Discussion

The CONFIDENCE-trial is the first large randomised controlled trial comparing the effect of LUS-guided deresuscitation to deresuscitation without LUS on clinically relevant outcomes in invasively ventilated ICU patients. The two strategies that are compared in this trial are currently used in the ICU without high-level evidence supporting the superiority of either. Deresuscitation is increasingly seen as an essential step in providing fluid therapy [28]. Nonetheless, it is unclear when, and in whom, to commence deresuscitation. In the quest for a modality to guide deresuscitation, LUS shows promising results and has many potential advantages compared to other modalities. Therefore, an adequately powered and methodologically sound RCT is warranted to evaluate the performance of LUS in guiding deresuscitation.

One recently published small RCT of LUS-guided deresuscitation in 176 surgical ICU patients found no differences in CFB nor an effect on 28-day or 90-day mortality when daily LUS was compared to routine care [29]. However, this study had several limitations. The authors planned to include 500 patients to obtain sufficient power, but this became unfeasible in the light of the SARS-CoV-2 pandemic. Furthermore, the authors hypothesised that the observed lack of difference in CFB might be caused by Enhanced Recovery after Surgery protocols that call for close monitoring of CFB and targeting a neutral or negative CFB. For non-surgical patients, such protocols do not exist, and these patients may thus benefit more from LUS-guided deresuscitation.

Major strengths of the current study include the large sample size, multicentre approach, and pragmatic protocol design. LUS is performed daily in the morning which is in line with current clinical practice. Ultrasound examinations are increasingly seen as a modern addition to physical examination [16]. For that reason, LUS should be quick to perform and standardised. In this study, we use a standardised 12-region LUS protocol, which is currently the best validated LUS monitoring protocol. Other strengths consist of the inclusion of a heterogeneous patient population and operating in both academic and community hospitals. Finally, due to the strict stabilisation criteria and deferred consent procedure, deresuscitation can commence quickly once indicated.

An important limitation of our study is the possibility that LUS-guided deresuscitation could be beneficial in patients who have received large amounts of fluid resuscitation. A heterogeneous ICU population is enrolled into this study, and patients that do not receive large amounts of fluid during resuscitation are not excluded from the primary analysis. While this facilitates results that are generalisable to a large proportion of ICU patients, this RCT lacks power if LUS-guided deresuscitation is only favourable in those that have had significant fluid resuscitation. However, we expect most patients to have undergone at least some fluid administration, as this is a routine intervention in care for the critically ill. Furthermore, fluid therapy is commonly administered on the general ward, emergency department, or during transport, and consequently, it is not always clear how much fluid has been administered before study initiation. Another relevant limitation of this study is that we cannot blind the caregiver to the intervention. Lack of blinding can lead to detection and performance bias [30]. The risk of performance bias is mitigated through the use of strict weaning criteria [31]. To reduce detection bias, data analysis will be performed blinded for the intervention. Finally, potential limitations could arise from the deferred consent procedure. Since legal representatives are made aware of the allocation of an enrolled subject, it might be that they are more likely to provide consent for subjects in the intervention or control arm. Potential imbalance in consent per allocation group will be analysed in the final manuscript.

In conclusion, the CONFIDENCE-trial is a multicentre RCT that aims to analyse the effect of daily LUS examination to guide deresuscitation in critically ill invasively ventilated patients on VFD-28. It will provide valuable insight into deresuscitation strategies for a large proportion of ICU patients. If proven effective, LUS-guided deresuscitation could improve outcomes in some of the most vulnerable and resource-intensive patients in a manner that is non-invasive, easy to perform, and well-implementable.

## Trial status

The first patient was included on December 25, 2021. The current version of the study protocol is version 6, dated May 16, 2022. The estimated study completion date is December 2024.

## Supplementary Information

**Additional file 1: Supplement I. Supplemental figure 1.** Lung regions used for lung ultrasound examination. **A**. supine position. Each hemithorax is divided in to six regions: upper and lower parts of the anterior, lateral and posterior chest wall. **B**: prone position. Each hemithorax is divided in to six regions: upper and lower parts of the posterior, lateral and anterior chest wall. Adapted from Heldeweg et al. with permission of the author (1). **Supplement II.** Definitions of all endpoints. **Supplement III.** Standard Protocol Items: Recommendations for Interventional Trials (SPIRIT) 2013 Checklist. **Supplement IV.** A list of all data collected.

## Data Availability

The full trial protocol and informed consent materials are available on any reasonable request to the corresponding author. The final trial dataset and the code for statistical analysis will be accessible by non-commercial partners upon request. A full monitoring plan is obtainable on reasonable request.

## Abbreviations

CFB: Cumulative fluid balance
CONFIDENCE: Effect of lung ultrasOuNd-guided Fluid deresuscltation on Duration of vEntilation iN intensive Care unit patiEnts (CONFIDENCE-trial)
DSMB: Data safety monitoring board
eCRF: Electronic case report form
EDC: Electronic data capture
GCP: Good clinical practice
ICU: Intensive care unit
LUS: Lung ultrasound
MAP: Mean arterial pressure
METC: Medical ethics committee
VFD-28: Ventilator-free days and alive at day 28
RASS: Richmond agitation sedation scale
RCT: Randomised controlled trial
WMO: Dutch Medical Research Involving Human Subjects Act
