# Supplementary material for: Effect of lung ultrasound-guided fluid deresuscitation on duration of ventilation in intensive care unit patients (CONFIDENCE): protocol for a multicentre randomised controlled trial
Source: Trials. 2023 Mar 24;24:226. doi: 10.1186/s13063-023-07171-w (PMC10038369; doi:10.1186/s13063-023-07171-w)
Supplement: Supplementary file 1 — Additional file 1: Supplement I. Supplemental figure 1. Lung regions used for lung ultrasound examination. A. supine position. Each hemithorax is divided in to six regions: upper and lower parts of the anterior, lateral and posterior chest wall. B: prone position. Each hemithorax is divided in to six regions: upper and lower parts of the posterior, lateral and anterior chest wall. Adapted from Heldeweg et al. with permission of the author (1). Supplement II. Definitions of all endpoints. Supplement III. Standard Protocol Items: Recommendations for Interventional Trials (SPIRIT) 2013 Checklist. Supplement IV. A list of all data collected. [file 13063_2023_7171_MOESM1_ESM.docx]

Supplement I

Lung ultrasound is performed for 12-regions. Each chest wall can be divided in to six lung regions: upper and lower parts of the anterior, lateral and posterior chest wall as can be seen in the figure below.


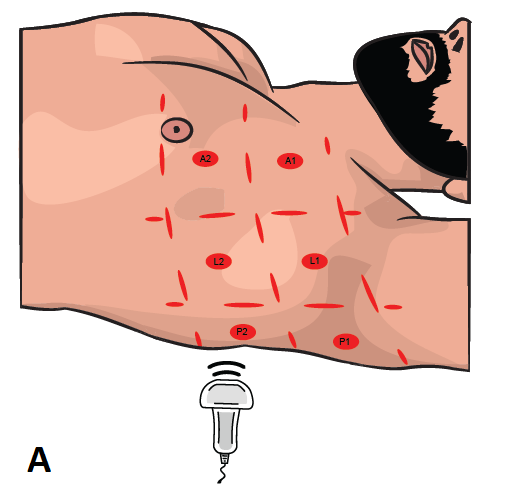

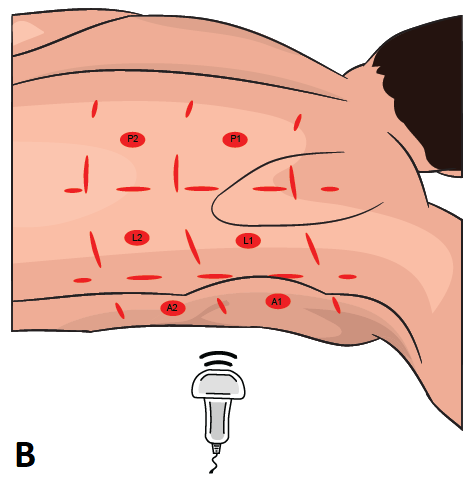


Supplemental figure 1: Lung regions used for lung ultrasound examination. A. supine position. Each hemithorax is divided in to six regions: upper and lower parts of the anterior, lateral and posterior chest wall. B: prone position. Each hemithorax is divided in to six regions: upper and lower parts of the posterior, lateral and anterior chest wall. Adapted from Heldeweg et al. with permission of the author (1).

For each lung region, points are allocated according to the worst ultrasound pattern observed:

- A-profile (0 points): No loss of lung aeration and presence of lung sliding with A-lines or <3 isolated B-lines;
- B1-profile (1 point): Moderate loss of lung aeration: ≥3 well defined B lines or <50 % of pleural surface affected;
- B2-profile (2 points): Severe loss of lung aeration: multiple coalescent B lines or ≥50 % of pleural surface affected;
- C-profile (3 points): Complete loss of lung aeration

Significant pleural effusion: >1 cm of pleural effusion in lateral regions and/or >2 cm of pleural effusion in posterior regions.

The lung ultrasound score is calculated as the sum of points of each lung region.

References:
1. Heldeweg, M. L. A., Lieveld, A. W. E., de Grooth, H. J., Heunks, L. M. A., Tuinman, P. R., & ALIFE study group (2021). Determining the optimal number of lung ultrasound zones to monitor COVID-19 patients: can we keep it ultra-short and ultra-simple?. *Intensive care medicine*, *47*(9), 1041–1043. https://doi.org/10.1007/s00134-021-06463-6

Supplement II

| **Domain** | **Measurement** | **Metric** | **Method of aggregation** | **Timepoint** |
| --- | --- | --- | --- | --- |
| Duration of invasive ventilation | Ventilator free-days | 28 minus days free of ventilation from the start of study. 0 in case of mortality | Median in control and intervention arm | 28 days |
| Duration of invasive ventilation | Duration of ventilation | Days of invasive ventilation from start of study | Median in control and intervention arm | 90 days |
| Mortality | 28 day all-cause mortality | Mortality, yes or no | Proportion in control and intervention arm | 28 days |
| Mortality | 90 day all-cause mortality | Mortality, yes or no | Proportion in control and intervention arm | 90 days |
| Mortality | 90 day ICU-mortality | Mortality during ICU admission, yes or no | Proportion in control and intervention arm | 90 days |
| Mortality | 90 day hospital mortality | Mortality during hospital admission, yes or no | Proportion in control and intervention arm | 90 days |
| Length of stay | ICU length of stay | Days of ICU admission from start of study | Median in control and intervention arm | 90 days |
| Length of stay | Hospital length of stay | Days of consecutive hospital admission from start of study | Median in control and intervention arm | 90 days |
| Fluid status | Cumulative fluid balance | Fluid output subtracted from fluid input. Cumulative from start of study. | Median in control and intervention arm | Days 1-7 after randomization |
| Fluid status | Cumulative fluid balance | Fluid output subtracted from fluid input. Cumulative from start of study. | Median in control and intervention arm | Days 1-7 after first lung ultrasound examination |
| Biomarker for hypoperfusion | Serum lactate | Mean serum concentration of lactate in mmol/L over days 1 to 7 | Median in control and intervention arm | Days 1-7 after study start |
| Respiratory failure | Reintubations | Incidence of intubation after initial extubation | Proportion in control and intervention arm | 28 days |
| Auxiliary therapies | Chest drain placement | Incidence of placement of intra-pleural drainage tubes | Proportion in control and intervention arm | 28 days |
| Auxiliary therapies | Use of invasive hemodynamic monitoring | Incidence of placement pulmonary artery catheter and pulse index continuous cardiac output devices. | Proportion in control and intervention arm | 28 days |
| Auxiliary therapies | Use of chest X-ray | Total number of chest X-rays performed per admission day | Median in control and intervention arm | 28 days |
| Possible harms associated with deresuscitation | Atrial fibrillation | Occurrence of atrial fibrillation as a clinical diagnosis by the treating physician | Proportion in control and intervention arm | 28 days |
| Possible harms associated with deresuscitation | Acute Kidney injury | Incidence of acute kidney injury according to KDIGO guidelines and only KDIGO stadium ≥ 2 | Proportion in control and intervention arm | 28 days |
| Possible harms associated with deresuscitation | Hypernatremia | Incidence of serum natrium concentration above 150 mmol/L at one or more study days | Proportion in control and intervention arm | 28 days |
| Quality of life | EQ-5D-5L | Total score on EQ-5D-5L of patients alive at day 28 | Median in control and intervention arm | 28 days |

Supplement III

|  |  | **Reporting Item** | **Page and Line Number** | **Reason if not applicable** |
| --- | --- | --- | --- | --- |
| **Administrative information** | | | | |
| Title | [#1](https://www.goodreports.org/reporting-checklists/spirit/info/#1) | Descriptive title identifying the study design, population, interventions, and, if applicable, trial acronym | 1, 1-4 |  |
| Trial registration | [#2a](https://www.goodreports.org/reporting-checklists/spirit/info/#2a) | Trial identifier and registry name. If not yet registered, name of intended registry | 2, 70-71 |  |
| Trial registration: data set | [#2b](https://www.goodreports.org/reporting-checklists/spirit/info/#2b) | All items from the World Health Organization Trial Registration Data Set | 2, 70-71  15, 393-394 11, 294-295 1, 40 1, 1-4 2, 55 2, 44 2, 47-49 5,123-133 2, 51-53 14, 369 2,55 14 ,368-370 8, 212-220 |  |
| Protocol version | [#3](https://www.goodreports.org/reporting-checklists/spirit/info/#3) | Date and version identifier | 14, 369-370 |  |
| Funding | [#4](https://www.goodreports.org/reporting-checklists/spirit/info/#4) | Sources and types of financial, material, and other support | 15, 393-394 |  |
| Roles and responsibilities: contributorship | [#5a](https://www.goodreports.org/reporting-checklists/spirit/info/#5a) | Names, affiliations, and roles of protocol contributors | 1, 5-37  15, 389-391 |  |
| Roles and responsibilities: sponsor contact information | [#5b](https://www.goodreports.org/reporting-checklists/spirit/info/#5b) | Name and contact information for the trial sponsor | 11, 294-297 1,13 |  |
| Roles and responsibilities: sponsor and funder | [#5c](https://www.goodreports.org/reporting-checklists/spirit/info/#5c) | Role of study sponsor and funders, if any, in study design; collection, management, analysis, and interpretation of data; writing of the report; and the decision to submit the report for publication, including whether they will have ultimate authority over any of these activities | 11, 294-297 15, 393-395 |  |
| Roles and responsibilities: committees | [#5d](https://www.goodreports.org/reporting-checklists/spirit/info/#5d) | Composition, roles, and responsibilities of the coordinating centre, steering committee, endpoint adjudication committee, data management team, and other individuals or groups overseeing the trial, if applicable (see Item 21a for data monitoring committee) | 11, 294-298 & 308-312 |  |
| **Introduction** |  |  |  |  |
| Background and rationale | [#6a](https://www.goodreports.org/reporting-checklists/spirit/info/#6a) | Description of research question and justification for undertaking the trial, including summary of relevant studies (published and unpublished) examining benefits and harms for each intervention | 4-5, 76-111 |  |
| Background and rationale: choice of comparators | [#6b](https://www.goodreports.org/reporting-checklists/spirit/info/#6b) | Explanation for choice of comparators | 4-5, 105-111 |  |
| Objectives | [#7](https://www.goodreports.org/reporting-checklists/spirit/info/#7) | Specific objectives or hypotheses | 5, 113-118 |  |
| Trial design | [#8](https://www.goodreports.org/reporting-checklists/spirit/info/#8) | Description of trial design including type of trial (eg, parallel group, crossover, factorial, single group), allocation ratio, and framework (eg, superiority, equivalence, non-inferiority, exploratory) | 5, 119-121 |  |
| **Methods: Participants, interventions, and outcomes** | | | | |
| Study setting | [#9](https://www.goodreports.org/reporting-checklists/spirit/info/#9) | Description of study settings (eg, community clinic, academic hospital) and list of countries where data will be collected. Reference to where list of study sites can be obtained | 5, 121-122 |  |
| Eligibility criteria | [#10](https://www.goodreports.org/reporting-checklists/spirit/info/#10) | Inclusion and exclusion criteria for participants. If applicable, eligibility criteria for study centres and individuals who will perform the interventions (eg, surgeons, psychotherapists) | 5-6, 124-134 & 6,151-154 |  |
| Interventions: description | [#11a](https://www.goodreports.org/reporting-checklists/spirit/info/#11a) | Interventions for each group with sufficient detail to allow replication, including how and when they will be administered | 6-7, 135-178 |  |
| Interventions: modifications | [#11b](https://www.goodreports.org/reporting-checklists/spirit/info/#11b) | Criteria for discontinuing or modifying allocated interventions for a given trial participant (eg, drug dose change in response to harms, participant request, or improving / worsening disease) | 6-7, 167-186  6, 178-179 |  |
| Interventions: adherance | [#11c](https://www.goodreports.org/reporting-checklists/spirit/info/#11c) | Strategies to improve adherence to intervention protocols, and any procedures for monitoring adherence (eg, drug tablet return; laboratory tests) | 6, 145-148  6, 151-155  10, 266-271  11, 314-315 |  |
| Interventions: concomitant care | [#11d](https://www.goodreports.org/reporting-checklists/spirit/info/#11d) | Relevant concomitant care and interventions that are permitted or prohibited during the trial | 7-8, 181-214 |  |
| Outcomes | [#12](https://www.goodreports.org/reporting-checklists/spirit/info/#12) | Primary, secondary, and other outcomes, including the specific measurement variable (eg, systolic blood pressure), analysis metric (eg, change from baseline, final value, time to event), method of aggregation (eg, median, proportion), and time point for each outcome. Explanation of the clinical relevance of chosen efficacy and harm outcomes is strongly recommended | 8, 216-224  12, 315-317  Supplement II |  |
| Participant timeline | [#13](https://www.goodreports.org/reporting-checklists/spirit/info/#13) | Time schedule of enrolment, interventions (including any run-ins and washouts), assessments, and visits for participants. A schematic diagram is highly recommended (see Figure) | 9, 225-226 |  |
| Sample size | [#14](https://www.goodreports.org/reporting-checklists/spirit/info/#14) | Estimated number of participants needed to achieve study objectives and how it was determined, including clinical and statistical assumptions supporting any sample size calculations | 10-11, 275-280 |  |
| Recruitment | [#15](https://www.goodreports.org/reporting-checklists/spirit/info/#15) | Strategies for achieving adequate participant enrolment to reach target sample size | 9, 243-247 |  |
| **Methods: Assignment of interventions (for controlled trials)** | | | | |
| Allocation: sequence generation | [#16a](https://www.goodreports.org/reporting-checklists/spirit/info/#16a) | Method of generating the allocation sequence (eg, computer-generated random numbers), and list of any factors for stratification. To reduce predictability of a random sequence, details of any planned restriction (eg, blocking) should be provided in a separate document that is unavailable to those who enrol participants or assign interventions | 9, 250-252 |  |
| Allocation concealment mechanism | [#16b](https://www.goodreports.org/reporting-checklists/spirit/info/#16b) | Mechanism of implementing the allocation sequence (eg, central telephone; sequentially numbered, opaque, sealed envelopes), describing any steps to conceal the sequence until interventions are assigned | 9, 251-252 |  |
| Allocation: implementation | [#16c](https://www.goodreports.org/reporting-checklists/spirit/info/#16c) | Who will generate the allocation sequence, who will enrol participants, and who will assign participants to interventions | 9, 250-253 |  |
| Blinding (masking) | [#17a](https://www.goodreports.org/reporting-checklists/spirit/info/#17a) | Who will be blinded after assignment to interventions (eg, trial participants, care providers, outcome assessors, data analysts), and how | 9, 250-253 |  |
| Blinding (masking): emergency unblinding | [#17b](https://www.goodreports.org/reporting-checklists/spirit/info/#17b) | If blinded, circumstances under which unblinding is permissible, and procedure for revealing a participant’s allocated intervention during the trial |  | Caregivers are not blinded |
| **Methods: Data collection, management, and analysis** | | | | |
| Data collection plan | [#18a](https://www.goodreports.org/reporting-checklists/spirit/info/#18a) | Plans for assessment and collection of outcome, baseline, and other trial data, including any related processes to promote data quality (eg, duplicate measurements, training of assessors) and a description of study instruments (eg, questionnaires, laboratory tests) along with their reliability and validity, if known. Reference to where data collection forms can be found, if not in the protocol | 10, 257-265 |  |
| Data collection plan: retention | [#18b](https://www.goodreports.org/reporting-checklists/spirit/info/#18b) | Plans to promote participant retention and complete follow-up, including list of any outcome data to be collected for participants who discontinue or deviate from intervention protocols | 10, 266-271 |  |
| Data management | [#19](https://www.goodreports.org/reporting-checklists/spirit/info/#19) | Plans for data entry, coding, security, and storage, including any related processes to promote data quality (eg, double data entry; range checks for data values). Reference to where details of data management procedures can be found, if not in the protocol | 10, 257-265 & 11-12, 323-324 |  |
| Statistics: outcomes | [#20a](https://www.goodreports.org/reporting-checklists/spirit/info/#20a) | Statistical methods for analysing primary and secondary outcomes. Reference to where other details of the statistical analysis plan can be found, if not in the protocol | 11, 292-296 |  |
| Statistics: additional analyses | [#20b](https://www.goodreports.org/reporting-checklists/spirit/info/#20b) | Methods for any additional analyses (eg, subgroup and adjusted analyses) | 11, 300-304 |  |
| Statistics: analysis population and missing data | [#20c](https://www.goodreports.org/reporting-checklists/spirit/info/#20c) | Definition of analysis population relating to protocol non-adherence (eg, as randomised analysis), and any statistical methods to handle missing data (eg, multiple imputation) | 11, 293-296 & 298-288 |  |
| **Methods: Monitoring** | | | | |
| Data monitoring: formal committee | [#21a](https://www.goodreports.org/reporting-checklists/spirit/info/#21a) | Composition of data monitoring committee (DMC); summary of its role and reporting structure; statement of whether it is independent from the sponsor and competing interests; and reference to where further details about its charter can be found, if not in the protocol. Alternatively, an explanation of why a DMC is not needed | 12, 310-319 |  |
| Data monitoring: interim analysis | [#21b](https://www.goodreports.org/reporting-checklists/spirit/info/#21b) | Description of any interim analyses and stopping guidelines, including who will have access to these interim results and make the final decision to terminate the trial | 12, 303-304  12, 315-319 |  |
| Harms | [#22](https://www.goodreports.org/reporting-checklists/spirit/info/#22) | Plans for collecting, assessing, reporting, and managing solicited and spontaneously reported adverse events and other unintended effects of trial interventions or trial conduct | 12, 315-319 Supplement II |  |
| Auditing | [#23](https://www.goodreports.org/reporting-checklists/spirit/info/#23) | Frequency and procedures for auditing trial conduct, if any, and whether the process will be independent from investigators and the sponsor | 12, 320-324 |  |
| **Ethics and dissemination** | | | | |
| Research ethics approval | [#24](https://www.goodreports.org/reporting-checklists/spirit/info/#24) | Plans for seeking research ethics committee / institutional review board (REC / IRB) approval | 16, 431-437 |  |
| Protocol amendments | [#25](https://www.goodreports.org/reporting-checklists/spirit/info/#25) | Plans for communicating important protocol modifications (eg, changes to eligibility criteria, outcomes, analyses) to relevant parties (eg, investigators, REC / IRBs, trial participants, trial registries, journals, regulators) | 12, 325-327 |  |
| Consent or assent | [#26a](https://www.goodreports.org/reporting-checklists/spirit/info/#26a) | Who will obtain informed consent or assent from potential trial participants or authorised surrogates, and how (see Item 32) | 9, 238-241 |  |
| Consent or assent: ancillary studies | [#26b](https://www.goodreports.org/reporting-checklists/spirit/info/#26b) | Additional consent provisions for collection and use of participant data and biological specimens in ancillary studies, if applicable | 9, 236-237 | No biological data is collected |
| Confidentiality | [#27](https://www.goodreports.org/reporting-checklists/spirit/info/#27) | How personal information about potential and enrolled participants will be collected, shared, and maintained in order to protect confidentiality before, during, and after the trial | 10, 272-278 |  |
| Declaration of interests | [#28](https://www.goodreports.org/reporting-checklists/spirit/info/#28) | Financial and other competing interests for principal investigators for the overall trial and each study site | 16, 440-443 |  |
| Data access | [#29](https://www.goodreports.org/reporting-checklists/spirit/info/#29) | Statement of who will have access to the final trial dataset, and disclosure of contractual agreements that limit such access for investigators | 16, 429-430 |  |
| Ancillary and post trial care | [#30](https://www.goodreports.org/reporting-checklists/spirit/info/#30) | Provisions, if any, for ancillary and post-trial care, and for compensation to those who suffer harm from trial participation | 16, 434- 436 |  |
| Dissemination policy: trial results | [#31a](https://www.goodreports.org/reporting-checklists/spirit/info/#31a) | Plans for investigators and sponsor to communicate trial results to participants, healthcare professionals, the public, and other relevant groups (eg, via publication, reporting in results databases, or other data sharing arrangements), including any publication restrictions | 11, 279-282 |  |
| Dissemination policy: authorship | [#31b](https://www.goodreports.org/reporting-checklists/spirit/info/#31b) | Authorship eligibility guidelines and any intended use of professional writers | 10, 281-282 | No intended use of professional writers |
| Dissemination policy: reproducible research | [#31c](https://www.goodreports.org/reporting-checklists/spirit/info/#31c) | Plans, if any, for granting public access to the full protocol, participant-level dataset, and statistical code | 16, 429-430 |  |
| **Appendices** | | | | |
| Informed consent materials | [#32](https://www.goodreports.org/reporting-checklists/spirit/info/#32) | Model consent form and other related documentation given to participants and authorised surrogates | 16, 398 |  |
| Biological specimens | [#33](https://www.goodreports.org/reporting-checklists/spirit/info/#33) | Plans for collection, laboratory evaluation, and storage of biological specimens for genetic or molecular analysis in the current trial and for future use in ancillary studies, if applicable | 16, 437 |  |

It is strongly recommended that this checklist be read in conjunction with the SPIRIT 2013 Explanation & Elaboration for important clarification on the items. Amendments to the protocol should be tracked and dated. The SPIRIT checklist is copyrighted by the SPIRIT Group under the Creative Commons “[Attribution-NonCommercial-NoDerivs 3.0 Unported](http://www.creativecommons.org/licenses/by-nc-nd/3.0/)” license. This checklist can be completed online using https://www.goodreports.org/, a tool made by the EQUATOR Network in collaboration with Penelope.ai

Supplement IV

- Data collected on admission and within first 24 hours:
- Gender and age (male + years)
- Height and weight at admission (kg + cm)
- Relevant medical history
- Reason for ICU admission
- Reason for ventilation
- Cause of respiratory failure
- APACHE II score and SAPS II score
- Respiratory status, on admission, and every day until day :
- Intubation status (if extubated: time of extubation)
  - - If reintubated: specify reason why
- Tracheostomy status (if tracheostomized: time of tracheostomy)
- Invasiveness of ventilation (invasive, non–invasive, or intermittent ventilation via tracheostomy, use of HFNO)
- Respiratory parameters every day at a fixed time point until cessation of ventilation:
- Peripheral oxygen saturation (%)
- End–tidal fractions of CO2 (kPa)
- PaO2 (kPa)
- PaCO2 (kPa)
- Arterial bicarbonate (mmol/L)
- Arterial pH
- Arterial base excess (mmol/L)
- Mechanical ventilation parameters every day until day 7 at a fixed time point
- Tidal volume (ml + ml/kg PBW)
- Respiratory rate (breath/minute)
- Level of positive end–expiratory pressure (cmH_2_O)
- Peak and plateau pressures, or level of pressure support (level above PEEP, and maximal airway pressure, cmH_2_O)
- Inspiration to expiration ratio
- Inspired oxygen fraction (%)
- Minute volume (liters/minute)
- Pulmonary compliance (ml/ cmH_2_O)
- Every day at a fixed time point until day 28, and on day 90:
- Location of patient, (in ICU, hospital, other facility, or home) and
- Life status (alive or deceased)
- Every day until day 28 or discharge from ICU, whatever comes first:
- Use of pulmonary artery catheter and or PiCCO (yes or no),
- Use of chest-X-ray and or chest-CT (yes or no)
- Atrial fibrillation (yes or no)
- Acute kidney injury (yes or no)
- ARDS (yes or no)
- Twice daily until day 28 or discharge from ICU, whatever comes first:
- For intervention group: use of lung ultrasound (yes or no)
  - - If yes: lung ultrasound score
    - If not: specify reason why
- For control group: use of lung ultrasound (yes or no)
  - - If yes: specify reason why
- Clinical data, every day until day 28 or discharge from ICU, whichever comes first:
- Vital signs (heartrate and blood pressure)
- Glasgow Coma Scale (GCS)
- Transfusions of blood products (type and ml)
- Infusion of crystalloids (type and ml)
- Infusion of (artificial) colloids (type and ml)
- Infusion of noradrenaline (mg)
- Cumulative use of diuretics (type and mg)
- Cumulative fluid balance (ml)
- Cumulative urine output (ml)
- Days on Renal Replacement Therapy (RRT)
- Arterial lactate (mmol/L)
- Creatinine (umol/L)
- Urea (mmol/L)
- Sodium (mmol/L)
- Trombocytes (x10^9^/L)
- Sequential Organ Failure Assessment score (SOFA) score
  - At day 28:
- EQ-5D-5L
